# Prenatal and Postnatal Therapies for Down's Syndrome and Associated Developmental Anomalies and Degenerative Deficits: A Systematic Review of Guidelines and Trials

**DOI:** 10.3389/fmed.2022.910424

**Published:** 2022-07-05

**Authors:** Zinnat Hasina, Chi Chiu Wang

**Affiliations:** ^1^Department of Obstetrics and Gynaecology, The Chinese University of Hong Kong, Shatin, Hong Kong SAR, China; ^2^Li Ka Shing Institute of Health Sciences, The Chinese University of Hong Kong, Shatin, Hong Kong SAR, China; ^3^School of Biomedical Sciences, The Chinese University of Hong Kong, Shatin, Hong Kong SAR, China; ^4^Chinese University of Hong Kong-Sichuan University Joint Laboratory in Reproductive Medicine, The Chinese University of Hong Kong, Shatin, Hong Kong SAR, China

**Keywords:** Down's syndrome, prenatal therapy, postnatal therapy, congenital anomaly, clinical trials

## Abstract

Down's syndrome (DS) is the most common genetic disorder at birth. Multiple developmental abnormalities before birth and early onset of degenerative deficits after birth are features of DS. Early treatment for the manifestations associated with DS in either prenatal or postnatal period may improve clinical outcomes. However, information available from professional bodies and to communities is very limited. We carried out a systematic review and attempted meta-analysis of clinical trials for developmental abnormalities and degenerative deficits in DS. Only 15 randomized controlled trials (RCTs) in 995 (24 days to 65 years old) individuals with DS showed some improvement in cognitive disorders, development and growth, and musculoskeletal problem. However, each trial used different parameters and methods to measure various outcomes. RCTs of prenatal interventions in fetus with DS are lacking. The efficacy and safety of specific interventions in DS are still largely unknown. Proper counseling of the potential treatment for pregnant mothers who wish to continue their pregnancy carrying fetus with DS, and to health care professionals who take care of them are not adequate nowadays.

## Introduction

**Figure 1:**
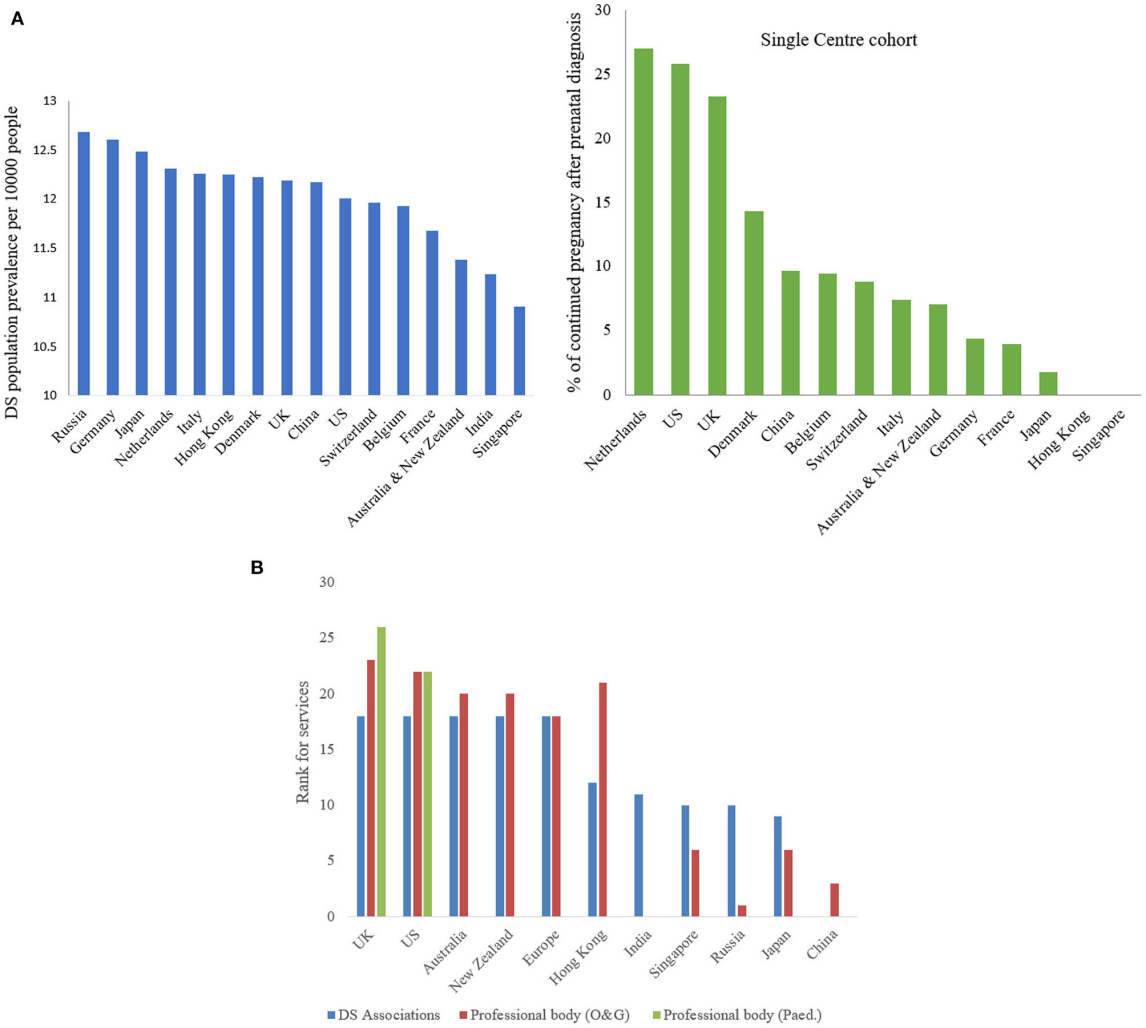
**(A)** Down syndrome population prevalence (blue) and % of continued pregnancy after prenatal diagnosis (green). Demonstrating extrapolated prevalence of DS population per 10000 people in different countries, the highest in Russia (12.68) and the lowest in Singapore (10.91) and the highest percentage of continued pregnancy after prenatal diagnosis in Netherlands (27.03) and the lowest in Japan (1.85). **(B)** Ranking of services provided by DS associations and professional bodies (O&G and pediatric sector). Showing the services provided by DS associations (blue in color) and professional bodies for O&G sector (red in color) and pediatric sector (green in color), only UK & US providing services in all sections.

Down's syndrome (DS) is the most commonly recognized numerical chromosomal disorder in live birth (1, 2). DS is caused by three copies (Trisomy 21) or partial translocation (Robertsonian translocation) of chromosome 21 due to chromosomal non-disjunction of unknown mechanism during meiosis. The incident rate is about one in 500–800 newborns throughout the world (3). Extrapolated prevalence of population with DS is around 10.91–12.68 per 10,000 people in different countries, the highest in Russia and the lowest in Singapore (Figure 1A) (4, 5). There are women in different countries whose pregnancies had confirmed DS by invasive prenatal diagnosis decided to continue pregnancy, the preference was the highest (27.03%) in Netherlands and the lowest (1.85%) in Japan (Figure 1A) (6–14).

Fetuses and individuals with DS present with variable spectrum and severity of developmental abnormalities before birth and early onset of degenerative deficits after birth (15–17). From diagnosis *in utero* to *ex-utero*, available clinical treatment is very little beyond current medical and genetic counseling (17). Most international DS associations only provide information and advice on prenatal screening, mental support, parenting skills, and specialist referral service; very few provide rehabilitation therapies but none in any recommendation of treatment. While learnt professional bodies of Obstetricians & Gynecologists and Pediatricians in most countries only offer health care professional for close monitoring and psychotherapy, but limited recommendation of any treatments for severe and life-threatened conditions (Supplementary Table S1 and Figure 1B).

Termination of the pregnancy could be one of the options for the mother and family (16), but not every parent carrying a fetus with DS would terminate the pregnancy, although the termination rate after prenatal diagnosis is high in some countries, 61–93% in US (7), 90% in UK (18) and 55.6–100% in China (13). Decide whether or not to continue the pregnancy rely on sufficient, adequate and updated medical evidence and ethical, unbiased and professional advice provided. If continue the pregnancy, the child may be born with hemodynamic disturbance due to cardiac defects, aspiration pneumonia and malnutrition due to duodenal atresia, and developmental delay and intellectual disability due to neurological retardation.

**Figure 2:**
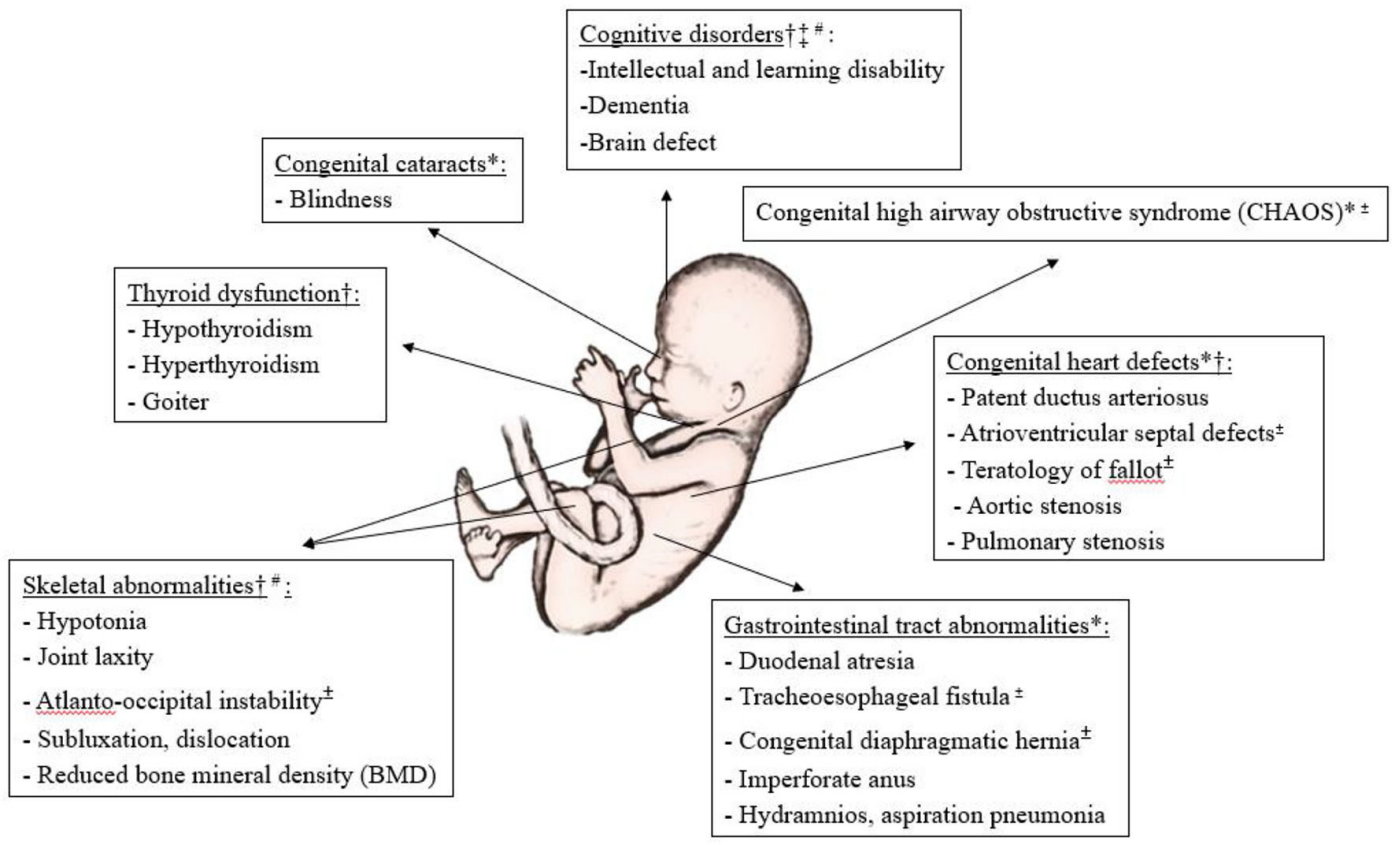
Illustration of common developmental anomalies and degenerative deficits in Down's syndrome, and the potential for prenatal and postnatal interventions. *Potential for prenatal surgical therapy, ^†^Potential for prenatal medical therapy, ^‡^Potential for prenatal gene therapy, ^±^Risk to life in utero or immediately after birth, ^#^Clinical trials available, Others if not indicated, mainly for postnatal intervention.

Some conditions, such as major atrioventricular septal defects, teratology of fallot, tracheoesophageal fistula and atlanto-occipital instability, may be extremely serious and even life-threatening *in utero* or immediately after birth (19) (Figure 2). Early intervention for the manifestations associated with DS in either prenatal or postnatal period may improve clinical outcomes. Before birth, some pioneering work on prenatal therapy offers new hope for treating the affected fetuses, and changing clinical decisions and management (20). These could provide an option for the management of life-threatening congenital malformations and could also improve the long-term health outcomes of individuals with DS (20, 21). However, the benefits of prenatal intervention and the potential risks to the fetus with DS are as yet unknown. After birth, it is well known that individuals with DS often benefit from speech, occupational and physical therapies (22, 23) to improve their communication and motor skills and to enhance posture and balance. Nevertheless, individuals with DS are also prone to develop other health problem (24). It is not yet clear whether any specific treatments to prevent or delay such problems are effective and safe.

In this study, we aimed to review and summarize available clinical trials for DS and relevant studies in order to update the prenatal and postnatal treatment for better counseling and potential management.

## Methods

### Study Design

This is a systematic review, the protocol was developed based on the Preferred Reporting Items for Systematic Review & Meta-analysis Protocols according to the PRISMA guidelines (Appendix A) (25), and was registered in the PROSPERO (CRD42018078933, available at www.crd.york.ac.uk/prospero).

### Sources

We carried out the literature search through interrogation of citations from PubMed, MEDLINE, EMBASE, and International Clinical Trial Registry Platform (ICTRP) from 1997 until December 2017. Reference lists of the included papers were also searched for additional potentially eligible trials. Keywords included “Down syndrome,” “prenatal therapy,” “postnatal therapy,” “congenital anomaly,” and “clinical trials.” The complete search strategy is shown in Appendix B.

### Inclusion Criteria

Studies included all published randomized controlled trials (RCTs) or cohort studies for any developmental abnormalities and degenerative deficits of DS before and after birth. Studies, which included participants with DS confirmed as Trisomy 21 or Robertsonian translocation, were eligible. If clinical trials for DS not available, clinical trials for similar developmental abnormalities and degenerative deficits as developed in DS were then recorded for reference.

### Exclusion Criteria

Studies were excluded if the articles related to animal studies, epidemiological studies, debate, commentary papers, imaging/laboratory studies, or review papers. Studies with no control group were also excluded.

### Data Extraction

Demographic data, including age, gender, sample size and diagnostic criteria; interventions methods, including pharmacologicals, surgery, speech/occupational/physiotherapy; outcomes measures, including methods, and assessment periods; and results, including therapeutics and side effects, were extracted independently by two authors using standardized forms. Discrepancies were resolved by discussion or adjudication of an independent advisor.

### Quality Assessment

We assessed risk of bias according to domains recommended in Cochrane Handbook for Systematic Reviews of Interventions (26). The risk of bias included sequence generation, allocation concealment, blinding, incomplete outcome data, selective reporting bias, and other sources of bias from compliance and baseline similarity. All domains were graded as low risk of bias, unclear, or high risk of bias (Supplementary Figure S1).

### Data Synthesis

Both positive and negative results were summarized. Side-effects of the intervention were also recorded. Mean and standard deviation (SD) or percentage of incidence and *p* values of the outcomes were recorded. Since there were no more than two trials used the same assessment methods to measure the same outcome, no meta-analysis was performed in this study.

## Results

### Trial Search Results

**Figure 3:**
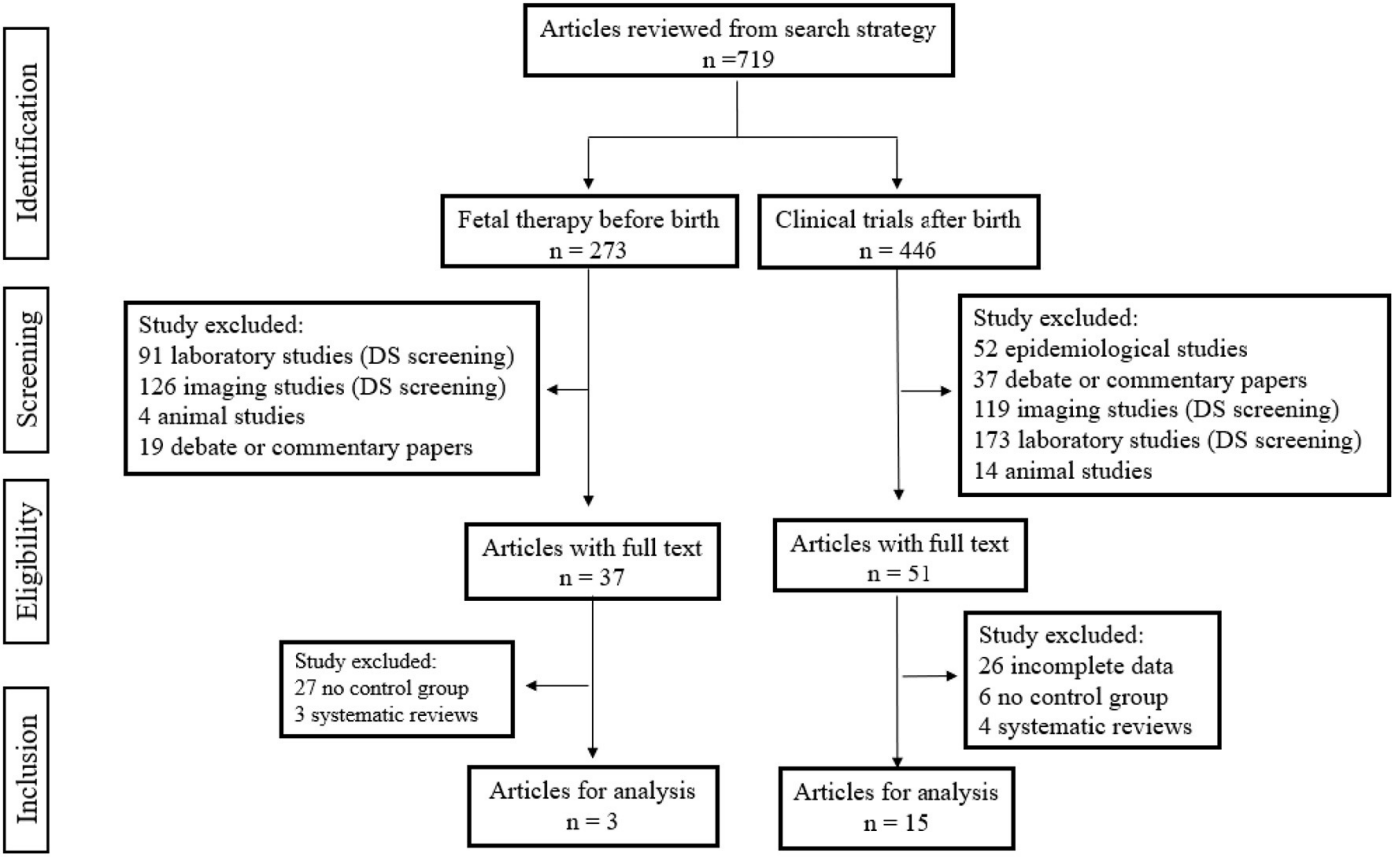
Flowchart. This is a diagrammatic representation of study selection process.

Study selection is summarized in Figure 3. For prenatal therapy of DS, 273 articles were screened. Of these, 126 imaging studies, 91 laboratory studies, four animal studies and 19 debate or commentary papers were excluded after assessing the titles and abstracts. Out of 33 full texts, 30 studies with no control group and review articles were excluded. At the end, no study was carried out on fetuses with DS, but three non-randomized clinical trials of similar congenital anomalies as DS were included. For clinical trials of DS after birth, 446 potential articles resulting from keyword searches were identified. After assessing the titles and abstracts, 52 epidemiological studies and 37 commentary papers were excluded, as well as 119 imaging studies, 173 laboratory studies and 14 animal studies. Fifty-one full text articles were evaluated for study eligibility, out of which 36 articles were excluded because of incomplete data, lack of a control group or because they were review articles. In the end, 15 RCTs were included.

### Clinical Trials Before Birth (Prenatal Therapy)

In our search of the literature, we were unable to find any clinical trial of prenatal therapy for DS fetuses. Only three clinical studies on similar congenital malformations to those associated with DS were found, and these were mainly surgical therapy for cardiovascular and respiratory conditions in fetuses without DS (Table 1).

**Table 1:** Prenatal therapy for congenital anomalies of Down's syndrome or similar anomalies associated with Down's syndrome.

**Publication**	**Study design**	**Congenital problem**	**Method**	**Subjects (DS or non-DS)**	**Sample size (cases/controls)**	**Outcome measures**	**Results**	**Mean ± SD/% (case vs control, *p* value)**	**Beneficial effects**	**Side effects**
**Surgical prenatal therapy**										
Tworetzky et al. (27)	Case report	Aortic stenosis	Fetal aortic valvuloplasty	Fetal aortic stenosis (non-DS)	20/4	1. maturity 2. AV length & dysfunction 3. MV inflow pattern 4. 4AA dimension	1.77% survived with improved aorti flow 2. 23% survived with biventricular circulation	1.5 ± 0.8 vs. 0.1 ± 0.6 *p* = 0.002 ± 1.3 vs. 0.7 ± 1.2 *p* = 0.02 2 ± 1 vs. 0.5 ± 1.1 *p* = 0.009	Prevent Progression	Postnatal interventions required
Tworetzky et al. (28)	Case-control	Pulmonary stenosis	Fetal pulmonary valvuloplasty	Fetal pulmonary atresia (non-DS)	10/15	1. Maturity 2. RV length 3. PV diameter	1.60% successful with Biventricular circulations after birth 2. 40% technically unsuccessful	+2.5 ± 0.9 vs. −4.2 ± 1.2 *p* = ≤ 0.05 +3.3 ± 1.1 vs. −5.1 ± 0.7 *p* = ≤ 0.05	Increase postnatal survival & biventricular circulation	40% failure rate challenging technique
Saadai et al. (29)	Cohort	Congenital high airway obstructive syndrome (CHAOS)	Ex *utero* tracheostomy and tracheoplasty	Fetal CHAOS (Congenital high airway obstructive syndrome) (non-DS)	4/8	1.Survival rate	1.100 vs. 100%	NA	Long term survival	Prognosis still poor

*DS, Down's syndrome; NA, not available; SD, standard deviation*.

For surgical prenatal therapy, there were three prenatal surgical interventions for congenital anomalies, mainly cardiovascular and pulmonary conditions associated with DS. However, no direct surgical procedure has been done on fetuses with DS in utero. The treatment success rates are about 100% (30) in ex-utero intrapartum treatment (EXIT) for congenital high airway obstructive syndrome (CHAOS) (30, 31) in humans, 70% (27) in fetal aortic valvuloplasty for fetal aortic stenosis (32, 33) in humans, and 60% (28) in fetal pulmonary valvuloplasty for fetal pulmonary stenosis (34, 35) in humans. These studies were assessed by procedural success, pregnancy outcomes and postnatal outcomes. The specific surgical operations may prevent the progression of disease (30), and increase postnatal survival rates (32, 33) and long-term survival (21). However, these interventions are risky for both mother and baby, and postnatal interventions may be required with fetal aortic/pulmonary valvuloplasty (27, 36). There are risks of premature rupture of membranes, preterm delivery, and intra-amniotic hemorrhage, and abdominal operative delivery may be required (34, 37). Overall, for surgical prenatal therapy 3 out of 3 studies (100%) obtained positive.

### Clinical Trials After Birth (Postnatal Therapy)

Among the 15 selected RCTs, common medical problems of DS in infants, children and adults were studied (Table 2). There were six clinical trials for cognitive disorders, two for development and growth, and seven for musculoskeletal disorders. Risk of bias in each of the included studies is shown in Supplementary Figure S1. All RCTs reported adequate methods of random sequence generation, and only two were unclear (45, 50). Most of the RCTs reported on allocation concealment, and only two were unclear (45, 52). Nine RCTs mentioned blinding of participants and personnel, and eight described adequate blinding of outcome assessment. Attrition bias was unclear in only one RCT (51). Risk of reporting bias was low in all but one RCT (52) and risks of other bias were unclear in most of the RCTs apart from four studies (40, 41, 43, 47). Overall, the risk of bias of the included studies was minimal.

**Table 2:** Postnatal therapy for individuals with Down's syndrome.

**Publication**	**Indication**	**Treatment approach**	**Treatment vs. control**	**Subjects (age/sex)**	**Sample size (cases/controls)**	**Outcome measures and methods**	**Positive results mean ± SD/% (case vs control, p value)**	**Negative results mean ± SD/% (case vs control, p value)**	**Beneficial effects**	**Side effects**
**Cognitive disorders**
Johnson et al. (38)	Cognitive impairment	Medical / Pharmacological therapy	Treatment: Donepezil PO 5 mg/d for the first 6 wks. and 10 mg/d for the remaining 6 wks. Control: Placebo	21–33 years /M & F	9/9	Cognitive functions assessments by Severe Cognitive Impairment Profile.	Language scores improved (90 ± 3.5 vs. 84 ± 8.3, *p* = 0.01)	No improvement in memory and attention (77 ± 8.4 vs. 76 ± 9.1, *p* = 0.9366)	Improved language performances.	None
Kondoh et al. (39)	Severe cognitive impairment	Medical / Pharmacological therapy	Treatment: Donepezil PO 3 mg/d for 24 wks.; Control: Placebo	32–58 years / F only	11/10	Chronological changes in psychological and motor functions by International Classification of Functioning, Disability and Health (ICF) scaling system.	Mental functions, voice and speech functions improved (10/11 vs. 0/10, *p* = <0.0001)	No improvement in movement related functions (2/11 vs. 0/10, *p* = 0.4762)	Improved Quality of life. Donepezil can use safely.	Soft stool Skin rash
Blehaut et al. (40)	Cognitive impairment	Medical / Pharmacological therapy	Treatment: Leucovorin PO 1 mg/kg/d for 12 months; Control: Placebo	3–30 months/ M & F	56/57	Developmental age by Brunet- Lezine scale.	Psychomotor development Improved (53.1 vs. 44.1%, *p* = 0.031)	NA	Improved cognitive functions.	None
Lott et al. (41)	Dementia	Medical / Pharmacological therapy	Treatment: Alpha-tocopherol 900IU, ascorbic acid 200 mg and Alpha-lipoic acid 600 mg/d PO for 2 years; Control: Placebo	45–55 years / M & F	27/26	Neuropsychological domains assessments by Brief Praxis Test (BPT).	NA	No effect on dementia (3.71 points 95%CI-4.81, 12.22, *p* = 0.39)	Antioxidant supplementation is safe	None
Mustafa Nachvak et al. (42)	Cognitive impairment	Medical / Pharmacological therapy	Treatment: Alpha-tocopherol 400IU/d and ALA 100 mg/d PO for 4 months. Control: Placebo	7–15 years / M & F	83/26	1. Oxidative stress by 8-hydroxy-2- deoxyguanosine (8OHdG) in urine 2.Thiobarbuturic acid reactive substances (TBARS) in serum.	Urinary 8OHdG concentrations decreased (−0.5 ± 0.6 vs. −0.1 ± 0.4, *p* = 0.04)	TBARS reduction is not significant.	Improved psychomotor and language development.	None
Marisa et al. (43)	Dementia	Medical / Pharmacological therapy	Treatment: Memantine PO 5-10 mg/d for 52 wks. Control: Placebo	> 40 years /M & F	72/74	Cognition function by dementia, attention, memory, and executive function scales (DAMES) score and adaptive behavior scale (ABS).	NA	No differences were observed (189.06 ± 79.73 vs. 195.07 ± 93.01, *p* = 0.75)	None	None
**Development and growth**										
van Trotsenburg et al. (44)	Delayed early development and growth	Medical / Pharmacological therapy	Treatment: Thyroxine PO 8 μg/kg/d for 2 years; Control: Placebo	20–30 days / M & F	90/91	1.Mental and motor development age at 24 months by Bayley Scales of Infant Development II. 2.Height and weight	Smaller delay in motor developmental age. (12.3 ± 2.1 vs. 13.0 ± 2.4, *p* = 0.015)	NA	Gained greater height and weight.	None
Marchal et al. (45)	Mental and motor development and growth	Medical / Pharmacological therapy	Treatment: Thyroxine PO 8 μg/kg/d for first 2 years of life; Control: Placebo	10.7 years / M & F	64/59	1. Mental and motor development by the Snijders-Oomen Nonverbal Intelligence test 2.5–7 (SON-R). 2. Communication skill by the Vineland Adaptive Behavior Scale (VABS). 3. Fine-motor coordination by the Beery-Buktenica Developmental test of Visual-Motor Integration fifth edition (Beery VMI). 4. HC, H and W	Height, weight and head circumference increased (50.5 ± 1.3 vs. 49.7 ± 1.6, *p* = 0.01)	No difference in mental or motor development, communication skill and fine motor coordination (4.4 ± 2.6 vs. 4.2 ± 2.5, *p* = 0.62)	Positive effects on growth.	NA
**Musculoskeletal problem**										
Carmeli et al. (46)	Lower limb muscle weakness	Physiotherapy	Treatment: Walking on the treadmill 10-15 min initially and gradually increases up to 45 min, thrice a week for 6 months; Control: Nonwalking	57–65 years /M & F	16/10	1. Dynamic balance and gait speed by Timed up and go (TUAG). 2. Knee extension 2. Flexion strength by medical isokinetic system (Biodex dynamometer).	1. Knee extension and flexion strength improved. 2. TUAG time is increased. (26.8 ± 4 vs. 57.8 ± 1, p = <0.01)	NA	1. Positive health benefits 2. Reducing risk of falls.	None
Gupta et al. (47)	Lower limb muscle weakness	Physiotherapy	Treatment: A specific exercise training programme, thrice a week for 6 weeks; Control: Usual therapy	7–15 years / M & F	12/11	1. Lower limb muscle strength by handheld dynamometer 2. Balance by the balance subscale of BruininksOseretsky Test of Motor Proficiency (BOTMP).	Strength of all the muscle groups and balance improved. (19.5, 16.25–24 vs. 9, 8.0–13.0; *p* = 0.001)	NA	1. Improved motor and gait functions. 2. May have psychological benefits.	None
Shields and Taylor (48)	Muscle weakness	Physiotherapy	Treatment: Progressive resistance training programme, twice a week for 10 weeks; Control: Usual activities	13–18 years / M & F	11/12	1.Muscle strength and physical activity by a timed stairs test 2.Grocery shelving task.	Lower limb muscle strength increased. (132 ± 50 vs. 97 ± 43, *p* = <0.05)	NA	Feasible, socially desirable and safe exercise.	Muscle soreness
Shield et al. (49)	Muscle weakness	Physiotherapy	Treatment: Progressive resistance training programme, twice a week for 24 weeks. Control: Social programme	14–22 years / M & F	34/34	1. Muscle strength by one-repetition maximum (1 RM) force generation tests. 2. Physical activity (measured as average vector magnitude activity) by RT3 activity monitor, lightweight accelerometer	1. Muscle strength increased at 11weeks 2. Physical activity increased at 24wks. (133.3 ± 59.5 vs. 101.3 ± 48.3, *p* = 0.03)	NA	Become stronger and more physically active.	Muscle soreness
Reza et al. (50)	Development of bone mineral density (BMD)	Physiotherapy and medical/ pharmacotherapy	Treatment: Weight bearing exercise for 45 min/day, 3 sessions/ week, and Dietary calcium PO 200mg / serving/d for 4 months; Control: No treatment	7–12 years / M & F	36/12	BMD by dual-energy X-ray absorptiometry.	Both exercise and calcium intake increased bone mineral density (0.0646 ± 0.4702 vs. 0.0451 ± 0.4168, *p* = 0.0001)	NA	Increase the bone mass.	None
Zubillaga et al. (51)	Vitamin D deficit	Medical/ pharmacological therapy	Treatment: Calcium 1 g/day and vitamin D 800IU/day PO for 1 year; Control: No treatment	28–46 years / M & F	12/11	1. BMD by dual-energy X-ray absorptiometry. 2. Biochemical tests related to the phospho-calcium metabolism.	No (2.36 ± 0.1 vs. 2.28 ± 0.1, *p* = >0.05)	NA	Correction of vitamin D and calcium deficiency	None
Ordonez et al. (52)	Muscle inflammation	Physiotherapy	Treatment: Motorized treadmill Three sessions /wk. for 10 wks. Control: No treatment	18–30 years / F only	11/9	1. Inflammatory markers by Plasma adipokine levels. 2. Fat mass percentage and distributions by bioelectrical-impedance analysis.	1. Plasma Leptin levels decreased 2. Fat mass % and WHR reduced. (45.7 ± 6.1 vs. 54.2 ± 6.7, *p* = 0.026)	NA	Reduced obesity	None

*PO, per oral; NA, not available; SD, standard deviation; BMD, bone mineral density; WHR, waist hip height ratio*.

For cognitive disorders, two studies (38, 39) showed that mental and speech functions were improved by oral administration of Donepezil, but no improvement in memory and attention was assessed by the Severe Cognitive Impairment Profile and the International Classification of Functioning, Disability and Health (ICF) scaling system. Another three studies (40–42) showed that oral administration of antioxidants (such as Alpha-tocopherol) and Leucovorin for cognitive impairment and dementia improved psychomotor development but was not effective for dementia assessed by the Brief Praxis Test (BPT), 8-hydroxy-2-deoxyguanosine (8OHdG) level in urine, thiobarbuturic acid reactive substances (TBARS) in serum and the Brunet-Lezine scale. Another study showed no significant benefits for dementia assessed by the Dementia, Attention, Memory, and Executive function Scales (DAMES) score with oral Memantine treatment (43). Soft stool and skin rash were seen as adverse effects in treatment with Donepezil, but other trials showed no side-effects during the course of the trials.

For development and growth, two studies (44, 45) showed that early treatment with thyroxin for delayed mental development and growth assessed either by the Bayley Scales of Infant Development II or the Snijders-Oomen Nonverbal Intelligence test 2·5-7 (SON-R) had positive effects in individuals with DS. No adverse effects were seen during the period of intervention.

For musculoskeletal disorders, various exercises were used in five studies, and the result showed increased muscle strength and improved gait development. Minor side-effects of muscle soreness due to use of weight equipment were observed in all studies. Of the five studies, three (47–49) used specific exercise training programs for muscle strength and balance, and were assessed by a timed stairs test, a grocery-shelving task, a handheld dynamometer, and the balance subscale of Bruininks Oseretsky Test of Motor Proficiency (BOTMP). Another study (46) used a treadmill for balance and muscle strength and was assessed by Timed Up and Go (TUAG) and a medical isokinetic system (Biodex dynamometer), respectively. Weight-bearing exercises and dietary calcium were used in another study (50), for the development of bone mineral density, which was assessed by dual-energy X-ray absorptiometry. One trial (51), using oral calcium and vitamin D, assessed by biochemical tests/markers related to the phospho-calcium metabolism/bone remodeling, showed positive effects for correction of vitamin D and calcium deficiency but no therapeutic effects on the musculoskeletal system. One study (52) used a motorized treadmill to evaluate the therapeutic effect and was assessed by bioelectrical-impedance analysis of plasma adipokine levels and fat mass percentage and distribution. The results showed reduced inflammation through reducing plasma leptin levels, and reduced obesity. No side-effects were observed during the trials.

Overall, in the cognitive disorders group, four out of six trials (66·7%) in DS individuals obtained positive results, and only two trials (33·3%) showed negative results. In the development and growth group, two out of two trials (100%) in individuals with DS achieved positive results. For the musculoskeletal disorders group, six out of seven trials (85·7%) in individuals with DS showed positive results, with only one trial (14·3%) showing negative results.

## Comment

### Prenatal Therapy for Fetuses With DS

Prenatal therapy consists of a series of prenatal interventions performed on the affected fetus. The procedures are highly complex and are performed only if the prognosis of the fetus is poor, that is, if the fetus may not survive or may be severely handicapped without intervention (53–55). The possible prenatal interventions for DS include medical and surgical, and the other potential therapies are gene and stem cell therapy.

Proactive and prophylactic fetal medical treatment is helpful for a variety of diseases, such as cardiac arrhythmias (56), thyroid abnormalities (57), and fetal thrombocytopenia (58, 59). We did not identify any clinical trials that employed medical or pharmacological therapy in fetuses with DS, apart from in some animal studies. These studies showed that early pharmacotherapy may be able to improve brain defects and behavioral deficits in the mouse model with DS. One study showed that neurogenesis and cognitive behavior were fully restored by once-daily subcutaneous injection of fluoxetine into the Ts65Dn pregnant mice from early embryonic stage until delivery (60). Some minor risk of pulmonary hypertension had been observed as an adverse effect. Another study showed that prenatal medical therapy with NAPVSIPQ (NAP) and SALLRSIPA (SAL) prevents developmental milestone delay in the mouse model of DS through effects on N-methyle-D-aspartic acid and ν-aminobutyric acid receptors (61). Partial learning deficit had been noticed as an adverse effect. The next one was performed on transgenic mice overexpressing Dyrk1A for brain defects, which showed that epigallocatechin gallate could rescue brain volume by inhibiting the Dyrk1A gene (62). No side-effects were observed during the procedure. The last one was done on pregnant Ts65Dn mice for attentional dysfunction and showed that prenatal choline supplementation significantly improved cognitive function and emotional regulation (63). Kidney stones and unknown causes of death occurred in some cases during the treatment.

Prenatal intervention by surgery may involve either a direct operation on a fetus with a structural or placental defect (64, 65). Successful prenatal surgical intervention may save the life of the fetus, for example prenatal surgery to repair a myelomeningocele in *utero* or selective laser photocoagulation on the vessels of the placenta to treat twin-to-twin transfusion syndrome (66, 67). We did not identify any clinical trials specific for fetuses with DS, but found other clinical trials associated with similar congenital anomalies to those of DS (27–29). These procedures were technically achievable, although risky for both fetus and mother; the treatments were successful in promoting long-term outcomes after surgery. Although the results seem promising, the evidence is still not sufficient and whether offering the prenatal interventions for DS still need more clinical studies.

Gene therapy is a form of advanced molecular technology that includes adding one or more corrective genes to the genetic material of the individual's cells in order to treat or prevent a genetic disorder. Stem cell therapy, also known as regenerative medicine, is the use of stem cells to treat or prevent a disease or condition. With regard to gene therapies for the fetus, research shows that transferring genes to the developing fetus uses rapidly growing populations of stem cells, which are unreachable after birth. Prenatal intervention, such as gene therapy for intellectual disability, may prevent the development of severe manifestations of early-onset disorders (68, 69). However, there are some potential risk factors of prenatal gene therapy, such as vector toxicity, germline modification, developmental aberration and oncogenesis, not to mention the procedural risks for the fetus and mother. More vigorous study and research are needed before gene therapy can be used (70). The success of *in-utero* hematopoietic stem-cell transplantation will probably serve as the first step toward the management of congenital hematological, metabolic, and immunological diseases (71). Until now, neither prenatal gene therapy nor stem cell therapy had been tested in DS.

### Postnatal Therapy for Individuals With DS

Research shows that intervention in the postnatal period improves outcomes for DS individuals (24). However, strong evidence is needed. In this review, we included 15 eligible RCTs of medical/pharmacological therapy and/or physiotherapy for common medical problems in DS individuals. The most commonly studied treatments were medical/pharmacological therapy for cognitive disorders, development and growth, and physiotherapy for musculoskeletal disorders and infections. Donepezil, Alpha-tocopherol and Leucovorin were shown to be effective for cognitive impairment and dementia, while thyroxin promoted development and growth of individuals with DS. However, no differences were observed after using Memantine for dementia. The therapeutic effect of antioxidants for cognitive disorders in DS is still elusive. Walking on the treadmills, and combined weight-bearing exercise and dietary calcium, but not calcium and vitamin D alone, can improve muscular strength and balance, and bone mineral density in individuals with DS.

### Difficulties of Clinical Trial in DS

The population with DS is relatively small, and clinical presentations of individuals and fetuses with DS are heterogeneous. Phenotypic manifestations and the prognosis of DS are variable; not all individuals with DS have the same medical conditions or disabilities. Furthermore, compliance with the intervention may be low, and the complications of clinical conditions may be hard to distinguish from the side-effects of the intervention. Many of the prenatal interventions have rarely been performed, and the results are therefore inconsistent and cannot be guaranteed. Moreover, some patients are referred from different centers, which make it difficult to collect follow-up data and postnatal monitoring, as these may vary according to institutional practice.

### Counseling

Though prevalence of trisomies has increased over time, mainly because of advanced maternal age, the live birth prevalence of trisomies has remained comparatively stable due to frequent prenatal screening and following termination (72). Over the past 20 years, the aim of prenatal counseling for DS has changed from radical management to possible proactive intervention. Yet no definitive therapeutic recommendations and guidelines are available. By using non-invasive prenatal testing and chorionic villus sampling, DS can be detected as early as at eight weeks' gestation and at 11-12 weeks' gestation, respectively. There is an opportunity for therapeutic intervention within a 28-29-week time span (73). Every affected pregnant woman or couple has the right to be informed about the treatment options. Since the diversity of abnormalities differs from fetus to fetus, DS associations and professional bodies do not believe that DS alone is a justification for termination of pregnancy. Besides surgical and medical treatment, professional bodies help parents understand the risk of the disorder and prepare emotionally; and also guide the parents and family about the management of pregnancy to reach decisions to develop a plan for delivery and neonatal care (Supplementary Table S1). Appropriate counseling regarding early interventions and therapy, as well as education, mental and social support, can help with better decision-making for the affected mother. During the progress of the pregnancy, additional testing provided if available to look for other health issues for better pregnancy management. DS organizations in different countries play an important role by organizing pre-school rehabilitation facilities for patients with DS. Through this review we found that there are many treatment options for early intervention and rehabilitation both prenatally and postnatally. In before, there were options either termination or continuation of pregnancy with all known disabilities of DS. Nowadays, fetal therapy could be another option for pregnant women who wish to continue their pregnancy by the latest development of modern medical and scientific technologies. By virtue of better treatments and therapies, increasing lifespan, family, social and community supports, DS individuals are flourishing physically and mentally. DS Associations build awareness about DS through Buddy Walk and World DS Day to let individuals with DS participate and live cherished lives in the communities. Associations, professional bodies, instructors and social welfares look forward to prospective time ahead for better understanding of DS problems and management. Unfortunately, there are no conclusive recommendations as to whether prenatal and postnatal interventions can be beneficial.

## Conclusion

Appropriate counseling should be provided to affected pregnant women and their families concerning the problems of DS and the therapies available, in order for optimal well-informed decision-making on whether or not to intervene. Although individual studies showed supportive evidence that some therapies can be used in early intervention programmes and throughout a individual's life with DS to promote the greatest possible development and independence, prenatal and postnatal therapies using similar parameters and outcomes for a specific DS condition are lacking. Further knowledge and guidance on effectiveness and safety of specific therapies to treat the various medical problems of DS are urgently required.

## Data Availability Statement

The original contributions presented in the study are included in the article/Supplementary Material, further inquiries can be directed to the corresponding author.

## Author Contributions

ZH conducted the searches, performed the data extraction, analyses and data entry, and wrote the article. CW contributed to the conception and design of the work and revised the manuscript, supervised ZH throughout this study and provided final approval for publication.

## Funding

The work is partially supported by Food and Health Bureau, Health and Medical Research Fund (01120156), CUHK Direct Grants (2002.2.024 and 2010.1.051), and CUHK Li Ka Shing Institute of Health Sciences Fund (6901988), Hong Kong.

## Conflict of Interest

The authors declare that the research was conducted in the absence of any commercial or financial relationships that could be construed as a potential conflict of interest.

## Publisher's Note

All claims expressed in this article are solely those of the authors and do not necessarily represent those of their affiliated organizations, or those of the publisher, the editors and the reviewers. Any product that may be evaluated in this article, or claim that may be made by its manufacturer, is not guaranteed or endorsed by the publisher.
