# Supplementary material for: Prenatal and Postnatal Therapies for Down's Syndrome and Associated Developmental Anomalies and Degenerative Deficits: A Systematic Review of Guidelines and Trials
Source: Front Med (Lausanne). 2022 Jul 5;9:910424. doi: 10.3389/fmed.2022.910424 (PMC9294288; doi:10.3389/fmed.2022.910424)
Supplement: Supplementary file 2 [file Image_1.pdf]

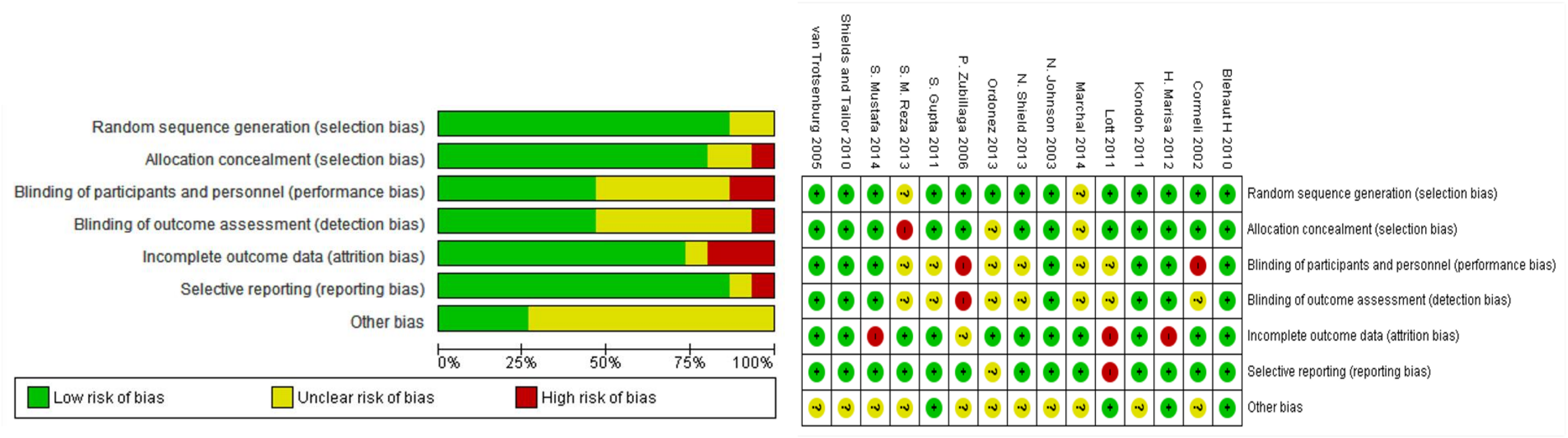

Figure S1. Risk of bias in included clinical trials for Down syndrome. The risk of bias included sequence generation, allocation concealment, blinding, incomplete outcome data, selective reporting bias, and other sources of bias. All domains were graded as low risk (green color), unclear (yellow color), or high risk (red color) of bias. Out of 15 RTCs 13 RCTs reported on allocation concealment, 9 RCTs mentioned blinding of participants and personnel, 8 RTCs described adequate blinding of outcome assessment, 14 RTCs cleared attrition bias, risk of reporting bias was low in 14 RTCs and risks of other bias were unclear in most of the RCTs apart from four studies. Overall, the risk of bias of the included studies was minimal.
