# Supplementary material for: Prenatal and Postnatal Therapies for Down's Syndrome and Associated Developmental Anomalies and Degenerative Deficits: A Systematic Review of Guidelines and Trials
Source: Front Med (Lausanne). 2022 Jul 5;9:910424. doi: 10.3389/fmed.2022.910424 (PMC9294288; doi:10.3389/fmed.2022.910424)
Supplement: Supplementary file 3 [file Table_1.docx]

| **Countries**  **Table S1. Services provided by Down’s syndrome Associations and Professional bodies** | **RUSSIA** | **EUROPE**  **(Germany, Netherlands, Italy, Denmark, Switzerland, Belgium & France)** | **JAPAN** | **HONG KONG** | **UK** | **CHINA** | **US** | **AUSTRALIA and NEW ZEALAND** | | **INDIA** | **SINGAPORE** | |
| --- | --- | --- | --- | --- | --- | --- | --- | --- | --- | --- | --- | --- |
| **Local DS Associations** | **Downside up (DSU)** | **European DS Association**  **(EDSA)** | **Japan DS Society**  **(JDSS)** | **Hong Kong DS Association**  **(HKDSA)** | **DS International**  **(DSi)** | **NA** | **National DS Society**  **(NDSS)** | **DS Australia**  **(DSA)** | **New Zealand DS Association**  **(NZDSA)** | **DS Federation of India**  **(DSFI)** | **DS Association (Singapore) DSA(S)** |  |
| **General information about DS** | Yes | Yes | Yes | Yes | Yes | NA | Yes | Yes | | Yes | Yes | |
| **Prenatal and postnatal counselling** | No | Recommend | Recommend (Postnatal only) | Recommend (Postnatal only) | Recommend | NA | Recommend | Recommend | | Recommend (Postnatal only) | NA | |
| **Genetic counselling** | No | Recommend | NA | NA | Recommend | NA | Recommend | Recommend | | NA | NA | |
| Antepartum | NA | Provide information & advice about prenatal screening | NA | NA | Provide information & advice about prenatal screening | NA | Provide information & advice about prenatal screening | Provide information & advice about prenatal screening | | NA | NA | |
| For mother |  |  |  |  |  |  |  |  | |  |  | |
| Postpartum | Mental support & parenting skill | Mental support & parenting skill | NA | Mental support & parenting skill | Mental support & parenting skill | NA | Mental support & parenting skill | Mental support & parenting skill | | NA | Mental support & parenting skill | |
| **Healthcare service** |  |  |  |  |  |  |  |  | |  |  | |
| **Table 1.Differences in advice of Professional bodies and Down’s syndrome Associations.**  Prenatal | No | provide information & referral specialist service | No | No | provide information & referral specialist service | NA | provide information & referral specialist service | provide information & referral specialist service | | No | No | |
| For baby |  |  |  |  |  |  |  |  | |  |  | |
| Postnatal | provide information & referral specialist service | provide information & referral specialist service | No | provide information & referral specialist service | provide information & referral specialist service | NA | provide information & referral specialist service | provide information & referral specialist service | | provide information & referral specialist service | provide information & referral specialist service | |
| Antepartum | No | No | No | No | No | NA | No | No | | No | No | |
| For mother |  |  |  |  |  |  |  |  | |  |  | |
| Postpartum | No | No | No | No | No | NA | No | No | | No | No | |
| **Therapies advice** |  |  |  |  |  |  |  |  | |  |  | |
| Prenatal | No | No | No | No | No | NA | No | No | | No | No | |
| For baby |  |  |  |  |  |  |  |  | |  |  | |
| Postnatal | No | No | No | No | No | NA | No | No | | Rehabilitation therapies | No | |
| **Helpline:** (toll-free helpline& email servic**e**) | NA | Yes | NA | Yes ( helpline service only) | Yes | NA | Yes | Yes | | NA | NA | |
| **Wellness & education** | Yes | Yes | Yes | Yes | Yes | NA | Yes | Yes | | Yes | Yes | |
| **Local & family support** | Yes | Yes | Yes | Yes | Yes | NA | Yes | Yes | | Yes | Yes | |
| **Awareness** | World DS Day (WDSD) | World DS Day (WDSD) | World DS Day (WDSD) | World DS Day (WDSD) | World DS Day (WDSD) | NA | Buddy Walk | World DS Day (WDSD) | | World DS Day (WDSD) | World DS Day (WDSD) | |
| **Regional or national Professional bodies (O&G sectors)** | **RSOG** | **EBCOG** | **JSOG** | **HKCOG** | **RCOG** | **CSOG** | **ACOG** | **RANZCOG** | | **ICOG** | **OGSS** | |
| **Pre-test counselling and information** | NA | Yes (only mother's age ≥ 35) | NA | Yes | Yes | NA | Yes | Yes | | NA | NA | |
| **Public screening tests** | NA | Still controversial | Yes | Yes | Yes | NA | No (private only) | Yes | | NA | No (private only) | |
| **Integrated Screening tests:** Combining first and second trimester screening tests results. | NA | Yes (only mother's age ≥ 35) | NA | Yes (For woman who wants to choose more sensitive or to reduce the false positive rate) | Yes | NA | Yes | NA | | NA | NA | |
| **Non-Invasive Prenatal Testing (NIPT):** Cell free DNA testing for fetal aneuploidy (cfDNA) | NA | Yes (Private test only) | Yes | Yes (Private test only | Yes | Yes (Private test only ) | Yes (Private test only) | Yes (Private test only) | | NA | Yes (Private test only) | |
| Screening quality assurance | NA | Yes | Yes | Yes | Yes | NA | Yes | Yes | | NA | NA | |
| **Diagnostic tests:**(Amniocentesis or CVS) | NA | Yes ( high risk case only) | Yes | Yes | Yes ( high risk case only) | Yes | Yes | Yes | | NA | Yes | |
| Continue pregnancy | NA | Continuation of pregnancy with frequent ultrasonographic monitoring of fetus | NA | Continuation of pregnancy with frequent ultrasonographic monitoring of fetus | Continuation of pregnancy with frequent ultrasonographic monitoring of fetus | NA | Continuation of pregnancy with frequent ultrasonographic monitoring of fetus | Continuation of pregnancy with frequent ultrasonographic monitoring of fetus | | NA | NA | |
| **Management guidelines** |  |  |  |  |  |  |  |  | |  |  | |
| Termination ofpregnancy(TOP) | TOP (only gestational age ˂22 weeks) | TOP (at any gestation for serious "fetal abnormalities" only) | TOP | TOP (only gestational age ˂24 weeks) | TOP (at any gestation for serious "fetal abnormalities" only) | TOP (up to 9 months of pregnancy) | TOP (in first trimester only) | TOP (no specific limitation) | | NA | TOP (only gestational age ˂24 weeks) | |
| Antepartum | NA | Information, advice & prenatal screening | NA | Information, advice & prenatal screening | Information, advice & prenatal screening | NA | Information, advice & prenatal screening (Private only) | Information, advice & prenatal screening | | NA | NA | |
| For mother |  |  |  |  |  |  |  |  | |  |  | |
| Postpartum | NA | Mental support | NA | Mental support | Mental support | NA | Mental support | Mental support | | NA | NA | |
| **Healthcare guidelines** |  |  |  |  |  |  |  |  | |  |  | |
| Prenatal | NA | Ultrasonographic follow-up | NA | Ultrasonographic follow-up | Ultrasonographic follow-up | NA | Ultrasonographic follow-up | Ultrasonographic follow-up | | NA | NA | |
| For baby |  |  |  |  |  |  |  |  | |  |  | |
| Postnatal | NA | Medical follow-up and provide information & advice | NA | Medical follow-up and provide information & advice | Medical follow-up and provide information & advice | NA | Medical follow-up and provide information & advice | Medical follow-up and provide information & advice | | NA | NA | |
| Antepartum | NA | Psychotherapy | NA | Psychotherapy | Psychotherapy | NA | Psychotherapy | Psychotherapy | | NA |  | |
| For mother |  |  |  |  |  |  |  |  | |  |  | |
| Postpartum | NA | No | NA | No | No | NA | No | No | | NA | NA | |
| **Therapies guidelines** |  |  |  |  |  |  |  |  | |  |  | |
| Prenatal | NA | Cardiac surgery for life-threatening heart defects. | NA | No | Cardiac surgery for life-threatening heart defects. | NA | Cardiac surgery for life-threatening heart defects. | Cardiac surgery for life-threatening heart defects. | | NA | NA | |
| For baby |  |  |  |  |  |  |  |  | |  |  | |
| Postnatal | NA | Surgery for cardiac and GI defects. | NA | Surgery for cardiac and GI defects. | Surgery for cardiac and GI defects. | NA | Surgery for cardiac and GI defects. | Surgery for cardiac and GI defects. | | NA | Surgery for cardiac and GI defects. | |
| **Genetic counselling** | NA | Yes | NA | Yes | Yes | NA | Yes | Yes | | NA | NA | |
| **Regional or national Professional bodies (Paediatric sectors)** | **RCWS** | **EAP** | **JPS** | **HKPS** | **RCPCH** | **CPS** | **AAP** | **APS & PSNZ** | | **IAP** | **SPS** | |
| **Guideline component for**  **screening and diagnosis** |  |  |  |  | **Prenatal, neonatal, and older** |  | **1 year and older** |  | |  |  | |
| **Information about DS & services available** | NA | NA | NA | NA | Yes, if diagnosed prenatally | NA | NA | NA | | NA | NA | |
| **Parents to be informed by senior paediatrician, medical assessment, & referral** | NA | NA | NA | NA | Neonatally | NA | NA | NA | | NA | NA | |
| **Haematologic abnormalities** | NA | NA | NA | NA | FBC & Hb every visit | NA | FBC & Hb annually | NA | | NA | NA | |
| **Cardiac abnormalities** | NA | NA | NA | NA | Assessment by ECHO & referral | NA | Assessment by ECHO & referral | NA | | NA | NA | |
| **Thyroid dysfunction** | NA | NA | NA | NA | TSH annually | NA | TSH annually | NA | | NA | NA | |
| **Obstructive sleep apnea** | NA | NA | NA | NA | Assess symptom annually | NA | Assess symptom annually. Polysomnogram by age 4 | NA | | NA | NA | |
| **Atlantoaxial instability** | NA | NA | NA | NA | Assess symptom at every visit | NA | Assess symptom at every visit | NA | | NA | NA | |
| **Hearing problems** | NA | NA | NA | NA | Hearing screen & referral annually | NA | Hearing screen & referral annually | NA | | NA | NA | |
| **Vision problems** | NA | NA | NA | NA | Vision assessment & referral annually | NA | Visional screening /referral  1-5 yrs. Annually,  6- 13 yrs. Every 2 yearly & every 3 yearly afterwards | NA | | NA | NA | |
| **Gastrointestinal problems** | NA | NA | NA | NA | Assess symptom annually | NA | Assess symptom annually | NA | | NA | NA | |
| **Developmental problems** | NA | NA | NA | NA | Early intervention referral by 3 years | NA | Early intervention referral by 3 years | NA | | NA | NA | |
| **Discussion about sexuality** | NA | NA | NA | NA | Assess yearly after at or after 12 yrs. | NA | Assess yearly after at or after 12 yrs. | NA | | NA | NA | |

DS = Down’s syndrome; TOP = termination of pregnancy; NA = not available; RSOG = Russian Society of Obstetricians and Gynaecologists; EBCOG = European Board & College of Obstetrics and Gynaecology; JSOG = Japan Society of Obstetrics & Gynecology; HKCOG = Hong Kong College of Obstetricians and Gynaecologists; RCOG = The Royal College of Obstetricians and Gynaecologists; CSOG = Chinese Society of Obstetricians and Gynaecologists; ACOG = American College of Obstetricians and Gynaecologists; RANZCOG = The Royal Australian and New Zealand College of Obstetricians and Gynaecologists; ICOG = Indian College of Obstetricians and Gynaecologists; OGSS = Obstetrical & Gynaecological Society of Singapore; RCWS = Russian Children's Welfare Society; EAP = European academy of pediatrics; JPS = Japan Pediatric Society; HKPS = Hong Kong Pediatric Society; RCPCH = The Royal College of Pediatrics and Child Health; CPS = Chinese Pediatric Society; AAP = American academy of pediatrics; APS & PSNZ = The Australian Pediatric Society & Pediatric Society of New Zealand; IAP = Indian academy of pediatrics; SPS = Singapore Pediatric Society.
